# Correction: Circulating levels of soluble Dipeptidylpeptidase-4 are reduced in human subjects hospitalized for severe COVID-19 infections

**DOI:** 10.1038/s41366-021-00988-y

**Published:** 2021-10-20

**Authors:** Kristina Schlicht, Nathalie Rohmann, Corinna Geisler, Tim Hollstein, Carina Knappe, Katharina Hartmann, Jeanette Schwarz, Florian Tran, Domagoj Schunk, Ralf Junker, Thomas Bahmer, Philip Rosenstiel, Dominik Schulte, Kathrin Türk, Andre Franke, Stefan Schreiber, Matthias Laudes

**Affiliations:** 1 Division of Endocrinology, Diabetes and Clinical Nutrition, Department of Medicine 1, University of Kiel, Kiel, Germany; 2 Institute of Clinical Chemistry, University of Kiel, Kiel, Germany; 3 Institute of Clinical Molecular Biology, University of Kiel, Kiel, Germany; 4 Division of Pneumology, Department of Medicine 1, University of Kiel, Kiel, Germany; 5 Interdisciplinary Emergency Center, University of Kiel, Kiel, Germany

**Keywords:** Obesity, Risk factors, Type 2 diabetes, Enzymes

Correction to: *International Journal of Obesity* 10.1038/s41366-020-00689-y, published online 21 September 2020

The article **Circulating levels of soluble Dipeptidylpeptidase-4 are reduced in human subjects hospitalized for severe COVID-19 infections**, written by Kristina Schlicht, Nathalie Rohmann, Corinna Geisler, Tim Hollstein, Carina Knappe, Katharina Hartmann, Jeanette Schwarz, Florian Tran, Domagoj Schunk, Ralf Junker, Thomas Bahmer, Philip Rosenstiel, Dominik Schulte, Kathrin Türk, Andre Franke, Stefan Schreiber and Matthias Laudes, was originally published electronically on the publisher’s internet portal on 21 September 2020 without open access. With the author(s)’ decision to opt for Open Choice the copyright of the article changed on 06 October 2021 to © The Author(s) 2021 and the article is forthwith distributed under a Creative Commons Attribution 4.0 International License, which permits use, sharing, adaptation, distribution and reproduction in any medium or format, as long as you give appropriate credit to the original author(s) and the source, provide a link to the Creative Commons licence, and indicate if changes were made. The images or other third party material in this article are included in the article’s Creative Commons licence, unless indicated otherwise in a credit line to the material. If material is not included in the article’s Creative Commons licence and your intended use is not permitted by statutory regulation or exceeds the permitted use, you will need to obtain permission directly from the copyright holder. To view a copy of this licence, visit http://creativecommons.org/licenses/by/4.0/

**Open Access funding enabled and organized by Projekt DEAL**

